# The impact of hospital competition on healthcare quality: evidence from China’s healthcare reform

**DOI:** 10.3389/fpubh.2025.1543884

**Published:** 2025-05-30

**Authors:** Huimin Liu, Junyan Jiang, Liangchun Yu, Xianpeng Liu

**Affiliations:** ^1^School of Economics and Management, Jiangxi University of Science and Technology, Ganzhou, China; ^2^School of Economics, Shandong University, Jinan, China; ^3^College of Architecture and Design, Jiangxi University of Science and Technology, Ganzhou, China

**Keywords:** hospital, competition, technical healthcare quality, non-technical healthcare quality, healthcare reform in China, sustainable development goals, SDG 3, good health and well-being

## Abstract

**Introduction:**

Hospital competition has been a common feature in healthcare reforms worldwide, yet scant attention has been paid to its impact on healthcare quality in China. This study aims to comprehensively evaluate the impact of hospital competition on healthcare quality under China’s healthcare reform.

**Methods:**

This study used multiple datasets comprising city-level and individual-level data of 21,974 individuals across 150 districts. We employed a two-way fixed effects model to estimate the impact of hospital competition on healthcare quality. To mitigate endogeneity concerns, we instrumented hospital competition with the inverse of the average distance and average travel time from patients to hospitals and performed the Two-Stage Least Squares methods.

**Results:**

This study showed a positive effect of hospital competition on both technical and non-technical healthcare quality. The impact is larger for primary hospitals and inpatient services; it is significantly positive for technical healthcare quality in public hospitals. Additionally, our findings implied that hospital competition affects healthcare income, which in turn affects non-technical healthcare quality, but healthcare income and demand jointly influence the relationship between hospital competition and technical healthcare quality.

**Conclusion:**

The finding provides new evidence of the impact of hospital competition on both technical and non-technical healthcare quality in China, highlighting a positive relationship that differs from some previous studies. This evidence offers valuable policy implications on hospital competition and also emphasizes the importance of considering the heterogeneity of hospitals and services in policy-making.

## 1 Introduction

Over the past few decades, hospital competition has played an increasingly significant role in the global healthcare market. Developed countries such as the United States and the United Kingdom are pioneers in advocating hospital competition, introducing policies promoting patient choice of providers, and incentivizing private surgical centers to compete with public hospitals (1, 2). With the global commitment to the Sustainable Development Goals (SDGs) which focus on good health and well-being, the study on the role of hospital competition as a key factor in affecting healthcare quality is important. However, researchers remain divided on the impact of hospital competition on healthcare quality (3, 4).

In China, the government has initiated a series of healthcare reforms to address the difficulty in accessing healthcare services, particularly those of high quality. An integral component of these reforms was fostering competition among healthcare providers which is designed to enhance healthcare efficiency and quality (5, 6). Beginning in 1980, private clinics were introduced to provide more healthcare services and incentivize competition between medical institutions. Subsequently, the New Healthcare Reform in 2009 underscored hospital competition as a cornerstone principle, leading to relaxed governmental regulations on public hospitals and an increased number of private hospitals by nearly 260%. This increasing number of hospitals affords patients greater options, and hospitals need to compete for patients to increase medical income.

There are different facilities and providers in the healthcare market, and their attributes may lead to interesting findings on the impact of hospital competition on healthcare quality. Following Liu (7), healthcare quality in primary and high-level hospitals is disparate and the quality is one of the provider factors pushing patients toward higher levels. It indicates different pressures on healthcare demand for different levels of hospitals as hospital competition increases. Patients’ attributes also matter. Patients with serious illnesses are particularly concerned about healthcare quality and prefer high-level hospitals, while patients with less severe conditions prefer primary hospitals (7). The hospital tends to provide patients with severe medical conditions with inpatient services and outpatient services for those with less serious conditions. Inpatient services typically involve a broader range of medical interventions and generate higher medical income. Patients with serious illnesses, who care more about quality, may affect hospitals’ strategies for outpatient and inpatient healthcare quality as competition increases.

Despite the extensive practice in China’s healthcare reform, limited attention has been given to the impact of hospital competition on healthcare quality in China compared to the abundant literature in developed countries (8–10). The diverse institutional backgrounds of healthcare systems between developed and developing countries may lead to different impacts of hospital competition on healthcare quality. China shares some common characteristics with several developing countries. These countries all have a large population, which exerts substantial pressure on their healthcare systems to improve healthcare quality. In these countries, public hospitals play a dominant role in providing basic medical services (11). Moreover, government policies actively stimulate hospital competition in these developing nations, while private hospitals are playing an increasingly significant part in their mixed healthcare delivery systems (12). Additionally, a portion of healthcare prices in these countries are subject to government regulation (13). Given these shared similarities, studying the impact of hospital competition on healthcare quality in China can offer valuable policy implications for other developing countries with comparable features. It can help them better understand how to optimize hospital competition through appropriate policies to improve healthcare quality. By analyzing China’s experience, these countries can learn from both the successes and challenges in promoting hospital competition, thus formulating more effective strategies to develop their healthcare systems.

The introduction of hospital competition in China has been relatively recent. While some previous studies have highlighted the significance of China’s healthcare reform institutional background in interpreting hospital competition (14, 15), systematic analyses are rare. Moreover, many existing studies in China rely on data from one specific city or province to estimate the effect of hospital competition (16), making it challenging to generalize regional findings to the entire country. Our research aims to bridge this gap between limited studies and abundant practices in China’s healthcare reform, providing insights for other countries contemplating the introduction of hospital competition.

This paper explores how hospital competition influences healthcare quality in China. We first analyze the institutional context of hospital competition in China. We then test the hypothesis on the effect of hospital competition on healthcare quality using a two-way fixed effects model with national city- and individual-level data. To address the endogeneity issue, we use the instrumental variable (IV) approach and instrument hospital competition with the inverse of average distance and average travel time from patients to hospitals. We also make analyses on the heterogeneity and mechanism of the effect of hospital competition on healthcare quality.

Measuring healthcare quality poses a longstanding challenge due to data limitations. Researchers have frequently used mortality or readmission rate as the proxy for healthcare quality, as lower mortality and readmission rates imply better treatment outcomes, and hence, better healthcare quality (17, 18). However, relying solely on mortality and readmission rates overlooks patient assessments of healthcare quality, which is essential for hospitals and health departments to evaluate healthcare quality comprehensively. We decompose healthcare quality into two components: technical healthcare quality and non-technical healthcare quality. We use whether the patient is readmitted within 1 year and patient satisfaction with healthcare quality as the proxy of technical healthcare quality and non-technical healthcare quality, respectively. These measures collectively capture healthcare quality.

Our findings indicate that hospital competition can decrease the probability of patients being readmitted within 1 year and increase patient satisfaction with healthcare quality, suggesting the positive association between hospital competition and healthcare quality, encompassing both technical and non-technical quality. Our robustness checks confirm the robustness of the result. In the heterogeneity analysis, our estimates reveal that hospital competition positively impacts technical healthcare quality for public hospitals, and it has a greater positive effect on both technical and non-technical healthcare quality for primary hospitals and inpatient services. Furthermore, our results suggest that hospital competition influences non-technical healthcare quality through healthcare income, but healthcare demand and income jointly influence the impact of hospital competition on technical healthcare quality.

Our study contributes to several bodies of literature. Firstly, it enriches the research on healthcare quality. To our knowledge, this paper is among the first to present the relationship between hospital competition and healthcare quality from both technical and non-technical perspectives, differing from the previous research studying healthcare quality from only one aspect (19, 20). Secondly, it extends regional findings from the existing literature in China by measuring hospital competition nationwide and providing empirical evidence with city- and individual-level data in the whole nation. Thirdly, the study expands the broader literature on hospital competition by examining its impact in a developing country context, offering policy insights for developing countries based on the evidence from China’s healthcare reform.

## 2 Institutional background

China’s healthcare system has evolved into a complex, multi-tiered structure. At its core lies a comprehensive public healthcare network that ensures accessible and affordable basic healthcare for the majority population. The healthcare system prioritizes hierarchical service provision: tertiary hospitals manage complex cases, secondary hospitals handle regional referrals, and primary hospitals address routine health needs (21). However, challenges remain. Medical resources are concentrated in urban tertiary hospitals and patients prefer high-level hospitals. Government-funded public hospitals play a pivotal role in delivering healthcare, especially in emergencies. Amid rising demand and resource constraints, the healthcare system increasingly integrates private-sector participation to enhance capacity (22).

China’s hospital sector is further stratified by ownership structures and operational objectives. Public hospitals, as system pillars, prioritize universal basic healthcare under price controls and regulations from the government (23). According to the China Health Statistics Yearbook, although public hospitals account for 32% of the total number of hospitals, they handle approximately 82% of outpatient visits and inpatient admissions. Yet public hospitals face inefficiencies in resource allocation. In contrast, private for-profit hospitals adopt market-driven strategies, leveraging flexible pricing and specialized services to attract patients willing to pay for shorter wait times and advanced technologies (24). Private for-profit hospitals make up around 44% of the total number of hospitals but only account for about 10% of outpatient visits and inpatient admissions. Private not-for-profit hospitals, typically established by charities or social enterprises, target underserved populations through subsidized care (24). Private not-for-profit hospitals constitute 24% of the total number of hospitals, with their share of outpatient visits and inpatient admissions being approximately 8%. The coexistence of public and private hospitals—both for-profit and nonprofit—creates a competitive landscape.

**Figure 1:**
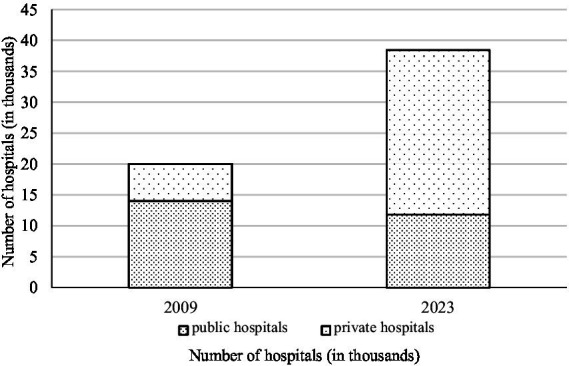
The number of private hospitals and public hospitals in 2009 and 2023. Data resource: the National Health Commission of China.

The Chinese government launched the New Healthcare Reform in 2009, and the number of hospitals has been steadily increasing, propelled by the hospital competition policy. According to the data from the National Health Commission of China, the number of hospitals in 2023 is 38,355 and it has increased by 92% since 2009. It indicates a heightened competition among hospitals and allows patients to select hospitals from a wide array of options (22). Consequently, the demand for the individual hospital decreases, implying that the hospital faces pressure due to the declining medical income. Figure 1 shows the number of private hospitals and public hospitals in 2009 and 2023. According to the data from the National Health Commission of China, the number of private hospitals in 2023 is 26,583, and it has increased by 280% since 2009. The number of public hospitals in 2023 is 11,772, and it has decreased by 17%. It suggests that private hospitals predominantly contribute to the burgeoning number of hospitals.

Public hospitals with longstanding government financial support in China have amassed abundant medical resources, attracting a large number of patients (25). However, the proliferation of private hospitals affords patients increased choices and allows patients to update their information on different types of hospitals. Besides, advertising efforts by private hospitals and the provision of differentiated healthcare services relative to public hospitals have the potential to alter patients’ preferences (26, 27). According to the data from the National Health Commission of China, the healthcare services provided by private hospitals have increased by 271% since the New Healthcare Reform, while those provided by public hospitals have only increased by 71%. This indicates a diminishing demand for services in public hospitals relative to private hospitals.

Notably, competition extends not only between public and private hospitals but also within both types. The dwindling financial subsidies mean that public hospitals rely more heavily on medical services and take medical services as their primary source of income (23). This intensifies the pressure on public hospitals as they face declining medical demand due to hospital competition. In China’s medical market dominated by public hospitals, private hospitals need to enhance their competitive advantages to attract patients from public and other private hospitals (14). That is, all hospitals need to compete for patients under the incentivization of competition.

And the competition between hospitals on healthcare prices is limited. The price of all services offered by private hospitals is determined by the market, while public hospitals are subject to price regulation for primary medical services, according to the healthcare pricing policy in China. Besides, hospitalization costs incurred at public hospitals are reimbursable under the basic health insurance program, whereas treatments at private hospitals remain excluded from such coverage. This reimbursement mechanism functions as a kind of subsidy to public institutions. Given this subsidized pricing structure, private hospitals face constraints in initiating price-based competition (28). Patients are relatively less sensitive to the healthcare price due to the reimbursement from medical insurance, and the attraction of low-price medical services for patients is limited (29). Consequently, hospitals are inclined to attract patients through non-price factors.

In addition to competing for equipment and technology, hospitals also engage in supply-side competition for scarce resources, such as skilled professionals. This competition drives strategic human resource management (SHRM) practices (30). Hospitals adopt human resource management (HRM) strategies such as competitive compensation, structured career development programs, and managerial optimization to attract and retain high-caliber medical professionals (31).

Paauwe’s framework highlights that HRM systems critically shape employee outcomes and affect organizational performance and patient care quality (32, 33). As the primary providers of healthcare services, skilled healthcare professionals’ competencies and work engagement directly influence care quality. An effective HRM system can enhance employees’ professional levels and job satisfaction through evidence-based training and incentive mechanisms (34). Physicians with advanced expertise deliver more accurate diagnoses and evidence-based treatments. Consequently, it can improve technical healthcare quality. At the same time, employees’ positive work attitudes and good service awareness also contribute to improving non-technical healthcare quality and enhancing patients’ perception of healthcare quality (35). For example, nurses’ compassionate care significantly increases patient comfort and satisfaction.

Competition reduces patient demand for individual hospitals, which incentivizes quality improvements. Rising expectations for higher quality healthcare drive this trend. However, information asymmetry complicates patients’ ability to assess quality. Hospitals tend to prioritize improving technical healthcare quality, such as physicians’ clinical expertise, because it is directly related to treatment outcomes and more objectively measurable (36). Nevertheless, improving non-technical quality, such as doctor-patient communication, is more challenging to quantify (37). Therefore, hospitals often prioritize technical quality improvements to stimulate healthcare demand.

Moreover, the downward pressure on medical demand caused by hospital competition increases financial pressure on medical income. Healthcare quality is positively correlated with service pricing, as patients are willing to pay premiums for superior care (38). Hospitals can charge higher prices whether they improve technical or non-technical quality. Although quality enhancements—such as HRM system investments—increase short-term costs, effective HRM systems reduce long-term operational costs (39). Therefore, in competitive markets, quality improvements can enhance hospital income.

Based on the foregoing analysis, we hypothesize that hospital competition has a positive effect on healthcare quality.

## 3 Data and methods

### 3.1 Data

The individual-level data comes from the China Health and Retirement Longitudinal Study (CHARLS). CHARLS is based on the Health and Retirement Study (HRS) and adopts multi-stage stratified probability-proportionate-to-size (PPS) sampling, collecting a high-quality nationally representative sample of Chinese residents ages 45 and older (40, 41). Before the data collection process in the CHARLS database, investigators undergo rigorous training covering questionnaire content, interview techniques, data entry procedures, and other aspects (42). CHARLS employs face-to-face structured questionnaire surveys. The baseline national wave of CHARLS was fielded in 2011, including 10,000 households and 17,500 individuals in 28 provinces, 150 counties/districts, and 450 villages/resident committees. The individuals are followed up every 2 years, and four follow-up surveys were performed in 2013, 2015, 2018, and 2020. The longitudinal tracking allows us to exploit within-individual variations in healthcare experiences after the new healthcare reforms. The modules on healthcare and insurance provide individual-level data on healthcare services, making it possible to test the hypothesis in micro scope. The CHARLS questionnaire includes the following modules: demographics, health status, and functioning, health care and insurance, community-level information, and so on. Especially, the module on healthcare and insurance provides individual-level data on healthcare services, making it possible to test the hypothesis above in micro scope. Due to the selection of key variables, we only use the data in 2015 and 2018 in this study.

The data on the number of hospitals, outpatients, and beds comes from the China City Statistical Yearbook, the provincial Health Statistical Yearbook, and the Bureau of Statistics (43). The China City Statistical Yearbook and the provincial Health Statistical Yearbook are published annually by the National Bureau of Statistics and the provincial Health Commission, respectively. China’s Top Hospitals by Outpatient Volume Ranking and Beds Ranking come from the official websites of Vistamed and the Institute of Asclepius Hospital Management (44, 45).

Healthcare quality is the degree to which health services for patients increase the likelihood of desired health outcomes, as defined by the World Health Organization (WHO). It can be conceptualized as a multidimensional construct, including both technical and non-technical (sometimes referred to as inter-personal or non-clinical) dimensions (46, 47).

Technical healthcare quality is the ability to achieve the improvement in health conditions that can be realized by current healthcare science and technology (46). It is measured by unplanned readmission within 1 year of discharge, a widely validated proxy for technical quality and clinical outcomes (48, 49). It is a binary variable, where 1 indicates that the patient was readmitted within 1 year and 0 denotes no readmission. This metric reflects the ability of hospitals to provide evidence-based care that prevents complications or recurrence, as emphasized in healthcare quality management by the National Health Commission of China.

Non-technical healthcare quality is the quality that is not directly related to healthcare technology or technical expertise but crucial for maintaining health (50). It includes interpersonal communication, facility environment, service efficiency, and so on. These dimensions directly shape patients’ subjective experiences of care, as they reflect how well healthcare services meet patients’ expectations for respectful interaction, accessible environments, and efficient processes (46). Non-technical healthcare quality is measured using patient satisfaction with healthcare quality, which is derived from the standardized survey assessing convenience, communication, and overall experience (51). Patient satisfaction with quality is an ordered variable varying from 1 to 5, where 1 represents a state of very dissatisfied, 2 indicates a state of somewhat dissatisfied, 3 corresponds to a neutral attitude, 4 signifies a state of somewhat satisfied, and 5 denotes a state of very satisfied. This metric aligns with frameworks recognizing patient-centered care as a critical determinant of healthcare quality, as patient satisfaction scores systematically capture the non-technical aspects of care that are central to this quality domain.

Many researchers measure hospital competition with the number of hospitals and the Herfindahl–Hirschman Index (HHI) (6, 8, 14, 52). However, relying solely on hospital numbers overlooks critical disparities in scale and service provision across hospitals. Similarly, calculating the HHI requires market share data, such as patient flow and bed capacity, which remain inaccessible at the national level due to privacy regulations and medical ethics constraints. To address these limitations, we employ a modified HHI framework that accounts for China’s unique healthcare system structure.

In China, hospitals are categorized into three tiers—primary, secondary, and tertiary—by the Ministry of Health, with each tier defined by standardized criteria for service capacity, facility size, and technical capabilities. The policy-driven structure enables us to measure hospital competition through the modified HHI. Specifically, we define market share as a hospital’s outpatient visits and beds within the healthcare market (53). Given the homogeneity of hospitals within the same tier, we first estimate market share using average outpatient volumes and bed counts for each tier. Considering that some large hospitals may deviate from the standard scale, we then incorporate publicly available rankings to refine their market share estimates. This measurement balances theoretical rigor with data feasibility, ensuring robust measurement of hospital competition in contexts where comprehensive market share information is unavailable. Following Yang et al. (54), the modified HHI based on the number of hospitals and outpatient flows across different tiers of hospitals is given by Equation 1:


(1) $$\mathrm{HHI}=\sum_{h}{\left(\frac{\bar{x_{h}}}{x}\right)}^{2}(N_{h}-n_{h})+\sum_{h}\sum_{l=1}^{n_{h}}{\left(\frac{x_{\mathrm{hl}}}{x}\right)}^{2}$$

where h is the tier of hospitals, x represents the number of outpatient visits. $\bar{x_{h}}$ is the average outpatient visits for h-tier hospitals. $N_{h}$ is the number of h-tier hospitals. $n_{h}$ represents the number of h-level hospitals listed in China’s Top Hospitals by Outpatient Volume Ranking, and $x_{\mathrm{hl}}$ represents the actual outpatient visits for these hospitals. Using a similar method, we can compute the HHI concerning the number of beds. We calculate the average HHI based on the number of outpatient visits and beds (55). Then, we use one minus this average HHI as a proxy for hospital competition, which is positively related to the level of hospital competition. We also use an alternative measure of competition, the number of hospitals, in robustness checks.

In this study, several control variables are used. The variable for income is log-transformed. Age is measured in years. Marital status is a binary variable, with 1 indicating that the individual is married and 0 indicating otherwise. Gender is also a binary variable, where 1 represents male and 0 represents female. Living in an urban area is a binary indicator, with 1 for those residing in urban areas and 0 for those in non-urban locations. Education is measured in years of schooling completed. The variable for having chronic diseases is binary, with 1 indicating the presence of chronic diseases and 0 indicating their absence. Enrolled by medical insurance is a binary variable, where 1 indicates the individual is covered by medical insurance and 0 indicates otherwise.

### 3.2 Methods

We use a two-way fixed effects model to test the hypothesis about the effect of hospital competition on healthcare quality, which shows by Equation 2:


(2) $$\mathrm{qua}l_{\mathrm{ijt}}=\beta_{0}+\beta_{1}\mathrm{com}p_{\mathrm{jt}}+\gamma X_{\mathrm{ijt}}+\mu_{i}+\delta_{t}+\varepsilon_{\mathrm{ijt}}$$

where i denotes the individual subscript, j represents the city index, and t indicates the year index. The dependent variable $\mathrm{qua}l_{\mathrm{ijt}}$ is the healthcare quality, and the independent variable $\mathrm{com}p_{\mathrm{jt}}$ shows the hospital competition. $X_{\mathrm{ijt}}$ is a vector of control variables. $\mu_{i}$ and $\delta_{t}$ show the unobserved time-invariant and individual-invariant characteristics, and $\varepsilon_{\mathrm{ijt}}$ is the error term.

Several endogeneity issues need addressing in our analysis. Firstly, omitted variable bias may arise from unobservable factors, potentially biasing our estimates. Secondly, there may be measurement errors in variables. Despite CHARLS being an authoritative database, it could introduce inaccuracies due to recording errors or respondents providing incorrect information.

Some researchers looked for instrumental variables to mitigate these biases and identify the effects of hospital competition accurately. One of the classic instruments for hospital competition is the function of distance from patients to hospitals. Kessler and McClellan (8) proposed a method for predicting the number of patients to calculate predicted hospital market share based on exogenous determinants of patient mobility, such as the travel distance for patients, rather than endogenous indicators. This approach is for measuring the level of hospital competition. Their study is part of a broader literature that predominantly uses the distance from patients to hospitals. This literature suggests that patients’ choice of hospitals is influenced by travel costs, with distance considered exogenous and determined by geographic factors independent of hospital quality (56–59). It implies that distance itself does not directly impact healthcare quality.

We instrument hospital competition with the inverse of average distance and average travel time from patients and hospitals. The shorter average distance and travel time increase competition among hospitals, as patients have more options within one specific region, and nearby hospitals compete for the same patient base. We take the inverse of the average distance and time to ensure that they vary in the same direction as the level of hospital competition. Such intensified competition may incentivize hospitals to enhance their healthcare quality to attract more patients. Therefore, the average distance and travel time to hospitals indirectly influence healthcare quality by affecting the degree of competition among hospitals.

The instruments are also required to be uncorrelated with omitted variables. A concern regarding this assumption might be that longer distances and travel time to hospitals could be linked to patients’ choices, reflecting the quality of healthcare services. Some patients are willing to travel longer distances and spend more time going to the hospital with high-quality healthcare services and high reputations. However, patient preferences for healthcare quality vary individually, and other patients may be unwilling to incur higher time and distance costs for high-quality services, considering emergencies, transportation expenses, and other indirect costs (60, 61). Higher-level hospitals can provide better healthcare services with more advanced medical facilities and specialized doctors (62). We estimate the relationship between hospital level and instrument variables and find that the estimates of the inverse of average distance and the inverse of average travel time from patients to hospitals are 0.258 (*p*-value = 0.412) and 0.161 (*p*-value = 0.112), respectively. Therefore, the instrument variables are insignificantly related to the choice of hospitals by patients at the 10% level. Our model also controls for the level of hospitals visited by patients to exclude the effect of patient choices for hospitals in the Section 4.2.

Another concern could be that patients with worse health may prefer to live nearer to the hospital, and the health status of the patient may affect the healthcare quality they receive. However, it seems implausible (60, 63). Patients with poorer health often have lower incomes and face higher medical expenses, while high-quality hospitals are typically located in urban centers or affluent areas where housing costs are high. Many patients may be unable to afford to live in these areas due to economic constraints. Moreover, good transportation infrastructure enables patients to access high-quality hospitals even if they do not reside nearby. We perform the regression analysis to examine the relationship between instrument variables and the patient’s health. We find that the estimates of the inverse of average distance and the inverse of average travel time to hospitals are 0.356 (*p*-value = 0.267) and 0.127 (*p*-value = 0.205), respectively. These results indicate that the instrument variables are insignificantly related to patient’s health at the 10% level. And our model also controls for the patient’s health status in the Section 4.2.

Besides, our model is over-identified and we can use the Hansen J statistic to test the validity of exclusion restrictions. And we conduct the estimation using the Two-Stage Least Squares (2SLS) method in the empirical analysis.

## 4 Results

### 4.1 Descriptive statistics

Table 1 provides detailed definitions of variables along with summary statistics, with 21,974 individuals included in the sample. Technical healthcare quality is only for the patients who received inpatient care in the past year and only 4,537 patients are included. The mean of technical healthcare quality is 0.315, suggesting that 31.5% of patients are readmitted within 1 year. Nontechnical healthcare quality is measured by patient’s satisfaction with healthcare quality which is an ordered variable, ranging from 1 to 5. The mean of nontechnical healthcare quality is 3.276, showing that average people are almost neutral to healthcare quality. The mean and standard deviation of hospital competition are 0.836 and 0.135, respectively. Besides, we control for a set of variables, including log (income), age, marriage, gender, urban, education, chronic disease, and insurance. Other information on control variables in Table 1 is not shown in the text because of space limitations.

**Table 1:** Descriptive statistics.

Variables	mean	sd	min	max	*n*
Technical healthcare quality	0.315	0.464	0	1	4,537
Nontechnical healthcare quality	3.147	1.302	1	5	21,974
Competition	0.836	0.135	0.154	0.957	21,974
Log (income)	7.217	3.483	0.637	17.425	21,974
Age	60.301	10.521	45	118	21,974
Married	0.836	0.352	0	1	21,974
Gender	0.490	0.514	0	1	21,974
Urban	0.257	0.457	0	1	21,974
Education	5.427	4.642	0	23	21,974
Chronic disease	0.538	0.465	0	1	21,974
Insurance	0.966	0.174	0	1	21,974

### 4.2 Estimation results

Table 2 presents the results regarding the impact of hospital competition on healthcare quality using the ordinary least squares (OLS) method. Column (1) shows the results for patient satisfaction, indicating a significantly positive association between hospital competition and patient satisfaction at the 10% level. Age and being married are negatively related to patient satisfaction at the 10 and 5% levels, respectively. Having medical insurance significantly increases patient satisfaction at the 10% level. The results in column (2) show the effect of hospital competition on the probability of patients being readmitted within 1 year, which presents a statistically negative coefficient at the 10% level. The presence of chronic diseases and medical insurance significantly increase the probability of patients being readmitted within 1 year at the 5% level. Being married is negatively related to patient readmission at the 5% level.

**Table 2:** Baseline regression: OLS method.

Variables	Satisfaction	Readmit
Competition	0.118* (0.069)	−0.125* (0.073)
Log (income)	0.002 (0.001)	−0.048 (0.035)
Age	−0.054* (0.031)	0.047 (0.030)
Married	−0.066** (0.031)	−0.167** (0.068)
Gender	−0.132 (0.093)	0.028 (0.152)
Urban	0.017 (0.019)	−0.023 (0.049)
Education	0.003 (0.004)	0.017 (0.025)
Chronic disease	−0.022 (0.032)	0.150** (0.086)
Insurance	0.520* (0.301)	0.262** (0.128)
Constant	−0.021 (0.031)	0.093 (0.156)
Year FE	Yes	Yes
Individual FE	Yes	Yes
*N*	21,974	4,537
Pseudo *R*^2^	0.186	0.362
Prob>chi2	0.000	0.000

Fixed effect (FE). Standard errors in parentheses are clustered at the individual level. *p < 0.10, **p < 0.05, ***p < 0.01.

The null hypothesis of the Durbin–Wu–Hausman test is strongly rejected at the 1% level, suggesting that hospital competition may be endogenous. Table 3 presents the 2SLS estimation results using the inverse of average distance and travel time from patients to hospitals as instruments. Column (1) shows that the estimated coefficient for hospital competition is significantly positive at the 1% level, indicating that hospital competition increases patient’s satisfaction with quality significantly. Column (2) shows hospital competition decreases the probability of patients being readmitted within 1 year at the 1% level.

**Table 3:** Baseline regression: 2SLS method.

Variables	Satisfaction	Readmit
Competition	1.228*** (0.372)	−2.073*** (0.518)
Other controls	Yes	Yes
Year FE	Yes	Yes
Individual FE	Yes	Yes
*N*	21,974	4,537
Pseudo *R*^2^	0.235	0.371
Prob>chi2	0.000	0.000
Hansen J statistic	0.257	0.409
Hansen J statistic (*p*-value)	0.612	0.522
IV 1st stage competition		
inv_avg_dist	1.047*** (0.136)	1.152*** (0.058)
inv_avg_time	1.547***(0.367)	1.625*** (0.214)
*F* statistic	303.892	289.826
Shea’s partial *R*^2^	0.348	0.293
Endogeneity test Chi^2^	33.991	25.632
Endogeneity test *p*-value	0.001	0.007

The inv_avg_dist and inv_avg_time mean the inverse of average distance and the inverse of average travel time from patients to hospitals, which are the instruments for hospital competition. Standard errors in parentheses are clustered at the individual level. *p < 0.10, **p < 0.05, ***p < 0.01.

The results of the 2SLS estimation are consistent with the OLS results generally, indicating that hospital competition can increase both technical and non-technical healthcare quality. However, there is a slight variation in the magnitude and statistical significance of the estimators between the OLS and 2SLS. The 2SLS estimation yields a larger absolute value of estimates, indicating a potential downward bias in the OLS results. Results from 2SLS specification are reported for all the models in Table 4 onward, given the inconsistency in OLS estimations.

**Table 4:** Impact of hospital competition on healthcare quality: controlling hospital level and patient’s health.

Variables	Satisfaction			Readmit		
	(1)	(2)	(3)	(4)	(5)	(6)
Competition	1.152*** (0.294)	1.121** (0.548)	1.213*** (0.284)	−1.508** (0.709)	−2.086*** (0.427)	−2.153*** (0.495)
Hospital level	Yes	No	Yes	Yes	No	Yes
Patient’s health	No	Yes	Yes	No	Yes	Yes
Other controls	Yes	Yes	Yes	Yes	Yes	Yes
Year FE	Yes	Yes	Yes	Yes	Yes	Yes
Individual FE	Yes	Yes	Yes	Yes	Yes	Yes
*N*	9,027	19,380	8,359	2,214	3,572	2,069
Pseudo *R*^2^	0.211	0.239	0.217	0.343	0.392	0.378
Prob>chi2	0.000	0.000	0.000	0.000	0.000	0.000
Hansen J statistic	0.248	0.265	0.234	0.421	0.417	0.382
Hansen J statistic (*p*-value)	0.617	0.608	0.625	0.516	0.520	0.537

Standard errors in parentheses are clustered at the individual level. *p < 0.10, **p < 0.05, ***p < 0.01.

The lower part of Table 3 presents the first-stage regression results for hospital competition. The *F* statistic in the first-stage regression is 303.892 in column (1) and 289.826 in column (2), which can be rejected with a probability value (prob>*F*) of 0.000. And the *F* statistic is larger than 10 for strong instruments. Shea’s partial *R*^2^ in column (1) and column (2) equals 0.348 and 0.293, respectively. The estimates of instruments are significantly positive at the 1% level in the first-stage regression. The evidence shows that instruments are significantly related to hospital competition.

The Hansen J statistic in column (1) and column (2) equals 0.257 and 0.409, respectively, and we fail to reject the null hypothesis of the test of overidentifying restriction at the 10% level. We also control for the hospital level and patient’s health which are omitted in the model above and may relate to the instruments, easing the omitted variables bias. The results are reported in Table 4, which shows similar estimates and significance with the results in Table 3, adding evidence that the instruments are exogenous. These tests above provide statistical evidence in favor of our IV specifications.

### 4.3 Robustness checks

There may be some threats to identifying the effect of hospital competition on healthcare quality. We then check the robustness of our estimates.

Firstly, we examine alternative measurements of key variables in Table 5. Patient satisfaction is an ordered variable ranging from 1 to 5 in the model, which may lead to the evaluation bias from patient’s subjectivity. We convert patient satisfaction into a binary variable with a value of 1 if the original value is larger than 2, otherwise, it equals 0. Column (1) presents the results, revealing a significant positive association between hospital competition and patient satisfaction at the 1% level.

**Table 5:** Robustness test I: alternative measurement for key variables.

Variables	(1)	(2)	(3)	(4)	(5)	(6)
	Satisfaction (dummy)	Length of stay	Satisfaction	Readmit	Satisfaction	Readmit
Competition	1.304*** (0.316)	0.492** (0.221)	1.006*** (0.214)	−1.442** (0.687)	3.145*** (0.730)	−4.128*** (0.526)
Other controls	Yes	Yes	Yes	Yes	Yes	Yes
Year FE	Yes	Yes	Yes	Yes	Yes	Yes
Individual FE	Yes	Yes	Yes	Yes	Yes	Yes
*N*	21,974	3,553	21,974	4,537	21,974	4,537
Pseudo *R*^2^	0.157	0.224	0.170	0.318	0.206	0.312
Prob>chi2	0.000	0.000	0.000	0.000	0.000	0.000

Standard errors in parentheses are clustered at the individual level. *p < 0.10, **p < 0.05, ***p < 0.01.

Following Moscelli et al. (10), we use the length of stay to measure non-technical quality in column (2). A longer length of stay in the hospital can indicate a higher volume of healthcare services provided, which may contribute to a lower readmission rate and potentially reflect better healthcare quality (48). The regression results demonstrate that the estimated coefficient for hospital competition is significantly positive at the 5% level. This suggests that heightened competition among hospitals can lead to an increase in the length of stay, potentially indicating improved healthcare quality.

We re-estimate the model with different measures of hospital competition in columns (3)–(6). Besides the HHI, another widely utilized measure for assessing the level of hospital competition is the number of hospitals (3). A greater number of hospitals generally signifies a higher degree of hospital competition. Following Lu and Pan (59), we use two alternative hospital competition measurements, namely the number of hospitals per 100,000 population in columns (3)–(4) and the number of new hospitals per 10,000 population in columns (5)–(6). The estimated coefficient of hospital competition is significantly positive at the 1% level in column (3) and column (5), suggesting a positive association between hospital competition and non-technical healthcare quality. Columns (4) and (6) present the results that hospital competition can decrease the probability of patients being readmitted at the 5 and 1% level, respectively, indicating that hospital competition is positively related to technical healthcare quality. Whether we use HHI or the number of hospitals to measure hospital competition, our results are robust, indicating that our findings are consistent across different measures of competition.

Secondly, we change the approach to define the hospital market in Table 6. In the baseline model, we use geopolitical boundaries to define the hospital market and it satisfies the Elzinga-Hogarty criteria in our data. However, there are 8.9% of patients seeking medical services in other cities. We redefine the hospital market based on the location of the hospitals visited by patients. The results in columns (1) and (2) show that hospital competition can increase both technical and non-technical healthcare quality.

**Table 6:** Robustness II: different hospital markets and covariates.

Variables	(1)	(2)	(3)	(4)
	Satisfaction	Readmit	Satisfaction	Readmit
Competition	1.502*** (0.312)	−2.106** (1.017)	1.273*** (0.364)	−2.375** (1.198)
Other controls	Yes	Yes	Yes	Yes
Year FE	Yes	Yes	Yes	Yes
Individual FE	Yes	Yes	Yes	Yes
Year*city FE	No	No	Yes	Yes
*N*	21,974	4,537	21,974	4,537
Pseudo *R*^2^	0.265	0.340	0.208	0.327
Prob>chi2	0.000	0.000	0.000	0.000

Standard errors in parentheses are clustered at the individual level. *p < 0.10, **p < 0.05, ***p < 0.01.

Thirdly, we explore whether the regression results differ with additional covariates in Table 6. We account for the potential endogeneity in the regressions by adding the interaction term between year and city. The results in columns (3) and (4) demonstrate that the estimated coefficients on hospital competition are consistent with the 2SLS results in Table 3.

These robustness checks provide further evidence supporting our main findings regarding the positive association between hospital competition and healthcare quality.

### 4.4 Heterogeneity analysis

The difference in medical resources between public and private hospitals, such as doctors and medical equipment, may lead to different healthcare quality with increased hospital competition. In Table 7, we estimate the impact of hospital competition on healthcare quality for public and private hospitals. For public hospitals, column (1) indicates a marginal and statistically insignificant positive correlation between hospital competition and patient satisfaction, while hospital competition decreases the probability of patients being readmitted within 1 year significantly at the 5% level in column (2). Columns (3) and (4) present regression findings for private hospitals, suggesting that hospital competition can enhance patient satisfaction and lower the probability of patients being readmitted, but the estimates are insignificant at the 10% level.

**Table 7:** Impact of hospital competition on healthcare quality by hospital ownership.

Variables	Public hospitals		Private hospitals	
	(1)	(2)	(3)	(4)
	Satisfaction	Readmit	Satisfaction	Readmit
Competition	0.203 (1.138)	−2.497** (1.215)	0.539 (1.107)	−0.028 (0.349)
Other controls	Yes	Yes	Yes	Yes
Year FE	Yes	Yes	Yes	Yes
Individual FE	Yes	Yes	Yes	Yes
*N*	5,115	2,616	1,181	1,608
Pseudo *R*^2^	0.117	0.207	0.143	0.245
Prob>chi2	0.000	0.000	0.000	0.000

All columns use outcomes for patients who saw a doctor in the hospital within the past year, based on CHARLS data. Standard errors in parentheses are clustered at the individual level. *p < 0.10, **p < 0.05, ***p < 0.01.

Then we estimate whether the impact of hospital competition on healthcare quality varies across different levels of hospitals. Results in Table 8 indicate that hospital competition significantly boosts patient satisfaction with healthcare quality and reduces the probability of being readmitted at the 1% level for both primary and high-level hospitals. Notably, the absolute value of the estimates for hospital competition is considerably larger in primary hospitals compared to high-level hospitals, suggesting that the impact of hospital competition on healthcare quality is larger in primary hospitals.

**Table 8:** Impact of hospital competition on healthcare quality by hospital level.

Variables	Primary hospitals		High-level hospitals	
	(1)	(2)	(3)	(4)
	Satisfaction	Readmit	Satisfaction	Readmit
Competition	7.960*** (2.095)	−8.824*** (0.173)	5.947*** (1.280)	−3.025*** (1.019)
Other controls	Yes	Yes	Yes	Yes
Year FE	Yes	Yes	Yes	Yes
Individual FE	Yes	Yes	Yes	Yes
*N*	5,297	2,237	1,636	1,744
Pseudo *R*^2^	0.242	0.280	0.194	0.236
Prob>chi2	0.000	0.000	0.000	0.000

Primary hospitals include county and district hospitals. High-level hospitals include regional, city, and provincial hospitals and those affiliated to a ministry level. All columns use outcomes for patients who saw a doctor in the hospital within the past year, based on CHARLS data. Standard errors in parentheses are clustered at the individual level. *p < 0.10, **p < 0.05, ***p < 0.01.

In Table 9, we explore the impact of hospital competition on healthcare quality for outpatient and inpatient services. Column (1) shows the estimated coefficient of hospital competition for outpatient services is significantly positive at the 1% level, indicating a positive impact on outpatient satisfaction with healthcare quality. The coefficient in column (2) is negative and significant at the 1% level, suggesting that hospital competition reduces the probability of revisiting for outpatients significantly. Columns (3) and (4) display the estimated coefficients for inpatient services, and the directions as well as statistical significance of the parameter estimated are consistent with the first two columns. It is noteworthy that the absolute value of the estimated coefficient of hospital competition for inpatient services surpasses that of outpatient services, implying a larger effect of hospital competition on inpatient healthcare quality.

**Table 9:** Impact of hospital competition on healthcare quality by the type of healthcare services.

Variables	Outpatient services		Inpatient services	
	(1)	(2)	(3)	(4)
	Satisfaction	Revisit	Satisfaction	Readmit
Competition	2.953*** (0.541)	−4.672*** (1.285)	12.739*** (4.109)	−8.980*** (0.573)
Other controls	Yes	Yes	Yes	Yes
Year FE	Yes	Yes	Yes	Yes
Individual FE	Yes	Yes	Yes	Yes
*N*	7,326	1,715	2,737	1,524
Pseudo *R*^2^	0.241	0.307	0.259	0.324
Prob>chi2	0.000	0.000	0.000	0.000

All columns use outcomes for patients who saw a doctor in the hospital within the past year, based on CHARLS data. Standard errors in parentheses are clustered at the individual level. *p < 0.10, **p < 0.05, ***p < 0.01.

### 4.5 Mechanism

After the heterogeneity analyses, we next explore the possible mechanisms that could have resulted in the effect of hospital competition on healthcare quality. Understanding how hospital competition alters patient behavior, thereby indirectly influencing the hospital’s choice of healthcare quality, is crucial. The theoretical analysis outlined above suggests that as competition increases, hospitals face the challenge of declining healthcare demand and income. As a result, hospitals are incentivized to enhance healthcare quality, which in turn allows them to attract more patients and potentially increase their income by adjusting healthcare prices in a competitive market. To explore the mechanism above, we use the number of patients and medical expenses as the proxy for healthcare demand and healthcare income for the hospital, respectively. Then we follow Liu et al. (64) and add the interaction term of hospital competition and patient satisfaction, as well as the interaction term of hospital competition and patients being readmitted within 1 year, into the model.

Table 10 presents the results for the mechanism. In column (1), the coefficient of the interaction term of hospital competition and patient satisfaction is positive but statistically insignificant at the 10% level; however, it is significantly positive at the 5% level in column (3). These results suggest that as hospital competition increases, non-technical healthcare quality is positively associated with medical expenses, leading to an increase in healthcare income, while exhibiting statistically insignificant effects on healthcare demand. In columns (2) and (4), the estimated coefficient of the interaction term of hospital competition and patients being readmitted is significantly negative at the 5% level, indicating that enhancing technical healthcare quality results in both increased healthcare demand and income as hospital competition increases.

**Table 10:** Mechanism analyses.

Variables	(1)	(2)	(3)	(4)
	Number of patients	Number of patients	Medical expense	Medical expense
Competition	−63.712 (43.723)	−3.092 (3.742)	0.322 (2.493)	−3.357 (2.547)
Satisfaction	−11.485* (6.719)		−0.767* (0.409)	
Competition*Satisfaction	11.325 (7.871)		0.975** (0.480)	
Readmit		18.890*** (5.896)		1.832** (0.926)
Competition*Readmit		−14.221** (6.959)		−1.998** (1.012)
Other controls	Yes	Yes	Yes	Yes
Year FE	Yes	Yes	Yes	Yes
Individual FE	Yes	Yes	Yes	Yes
*N*	6,523	3,780	6,989	3,272
*R* ^2^	0.136	0.244	0.143	0.269
Prob>*F*	0.000	0.000	0.000	0.000

Standard errors in parentheses are clustered at the individual level. *p < 0.10, **p < 0.05, ***p < 0.01.

These findings imply that hospital competition influences non-technical healthcare quality primarily through healthcare income, whereas its impact on technical healthcare quality operates through both healthcare demand and income.

## 5 Discussion

In this paper, we study whether hospital competition has a potentially positive effect on healthcare quality based on China’s healthcare reform. To clarify the effect, we present the hospital decision-making and patient choice under China’s healthcare reform and provide empirical evidence. Our results indicate that hospital competition can increase patient satisfaction and decrease the probability of patients being readmitted within 1 year, supporting the hypothesis that hospital competition is positively related to healthcare quality.

Understanding the role of hospital competition in influencing healthcare quality is crucial for the direction of China’s healthcare reform in the future. Based on the results, hospital competition leads to improved technical and non-technical healthcare quality, which indicates that hospital competition could serve as an effective method to address the difficulty in accessing high-quality healthcare services. And the government needs to make efforts to promote hospital competition in healthcare reforms.

Our finding that hospital competition has a positive effect on healthcare quality is consistent with findings from previous research conducted in Ghana (62) and the English National Health Service (65). In Ghana, healthcare is delivered through a mixed public-private system, where competition among providers is fostered under structures for regulated fees. Similarly, in England, the National Health Service operates with a predominantly public provision model, yet competition between public and private surgical centers is encouraged to improve quality. These settings share similarities with China’s healthcare system: all three rely on a public-dominated infrastructure, with government intervention in pricing mechanisms to ensure affordability. These parallels underscore a universal mechanism: under price regulation, hospitals tend to compete on non-price dimensions, such as service quality, to attract patients. This consistency indicates that regulatory frameworks that limit price-based competition can drive quality improvement in healthcare systems (66).

However, our results differ from those of Lin et al. (58), who reported that hospital competition had a mixed effect on healthcare quality in Shanxi province, China. We infer that the difference in findings results from the sample selection. In this study, we chose a representative sample covering diseases of varying severity. In contrast, Lin et al. (58) only studied patients with Acute Myocardial Infarction (AMI) and pneumonia. For these patients, the urgency of care restricts the elasticity of patient choice. Patients with different diseases show varying demand elasticities for healthcare services, which can influence hospitals’ incentives to compete on quality. Moreover, the concentrated hospital markets in Shanxi, as studied by Lin et al. (58), may dampen competition effects. In comparison, our national data capture variations in competitive intensity across China. Overall, our study offers a more generalizable view of the role of competition in China’s healthcare system, while the work of Lin et al. (58) emphasizes the importance of considering patient-specific factors in such analyses.

Our heterogeneity analyses reveal three critical patterns in China’s hospital competition, shaped by institutional hierarchies and market fragmentation. First, the effect of hospital competition on healthcare quality is different between public and private hospitals. Public hospitals leverage governmental subsidies to enhance technical quality, yet neglect non-technical aspects, while resource-constrained private hospitals struggle on both fronts due to insurance exclusion and patient distrust (25, 67). Second, competition exerts asymmetric pressure across hospital tiers. Primary hospitals, which face saturated markets and lower entry barriers, actively improve responsiveness to retain patients, whereas tertiary hospitals, protected by brand inertia and lax referral mechanisms, demonstrate minimal adjustments in quality (68). Third, competition-driven quality improvements concentrate on inpatient services, whereas outpatient services exhibit weaker responses. This divergence arises because inpatient care targets critically ill patients who prioritize observable clinical outcomes, and hospitals prioritize inpatient investments due to their income dominance (3, 58).

We find that hospital competition influences non-technical healthcare quality primarily through healthcare income, whereas its impact on technical healthcare quality operates through both healthcare demand and income. This difference can be attributed to the following reasons. Patients struggle to access accurate information for healthcare services due to asymmetric information, hence they tend to rely on technical healthcare quality which is easily perceived, such as the reputation of physicians and the number of advanced medical equipment. Moreover, technical healthcare quality is closely linked to disease treatment efficacy. The hospital can attract more patients and make more income by improving technical healthcare quality. Enhancing non-technical healthcare quality can elevate patient satisfaction with healthcare services, prompting patients to be more willing to pay higher fees, thereby increasing healthcare income for the hospital.

This study demonstrates three key strengths. First, it systematically evaluates the impact of hospital competition on both technical healthcare quality and non-technical healthcare quality, offering a comprehensive quality assessment absent from prior single-dimensional analyses. Second, the integration of national individual-level data from 28 provinces with the city-level competition index and instrumental variables, addresses endogeneity while ensuring generalizability beyond region-specific limitations. Third, the analysis of heterogeneous effects—particularly stronger quality improvements in primary hospitals and inpatient services—provides actionable evidence for designing targeted and differentiated competition policies in China’s healthcare system. Collectively, these contributions position the study to inform competition-driven reforms in developing countries with similar institutional contexts, particularly those that balance market forces with public healthcare provision.

Our study is subject to several limitations. Firstly, the individual-level data from CHARLS targets individuals aged 45 and older and is not exclusively designed for patients, which may impact the results. Access to the national clinical records data could potentially yield more robust estimates in future research. Secondly, the HHI we employ to measure hospital competition is based on the average market share for each level of hospitals, rather than the actual market share for each hospital. It may be an approximation for actual HHI, as the data on market share for all nationwide hospitals is unavailable and the scale of each level of hospital is similar. And we also use an alternative measurement, the number of hospitals, to show the robustness of the results. The actual market share can provide more information about hospital competition, although there is not a single, agreed-upon measure that is immune to every form of bias. Thirdly, it remains unclear how the impacts of the entry for hospitals may differ from the increasing choices of patients for hospitals, both of which incentivize hospital competition in healthcare reforms. Our analysis examined the association between healthcare quality and hospital competition which is measured with the information on hospitals only. Hence, it would be interesting to explore the different mechanisms of hospital competition on healthcare quality from both supply-side and demand-side perspectives in future research.

## 6 Conclusion

Hospital competition has become a prominent factor in healthcare reforms in recent years, driven by policymakers aiming to enhance healthcare delivery and quality. This study provides novel evidence on the impact of hospital competition on healthcare quality in China, encompassing both technical and non-technical healthcare quality. Integrating the national individual-level data from 28 provinces with the city-level competition index, we find that hospital competition significantly reduces the probability of patient readmission and enhances patient’s satisfaction with healthcare quality. These effects are amplified in primary hospitals and inpatient services, with public hospitals demonstrating marked improvements in technical quality. These results underscore how market pressures interact with institutional features. Resource-constrained primary hospitals adapt more dynamically to retain patients compared to higher-tier hospitals; furthermore, the reliance on income from inpatient services drives hospitals to achieve higher quality in inpatient services than in outpatient services, while public hospitals prioritize technical quality enhancement with governmental subsidies. Notably, our instrumental variables approach addresses endogeneity concerns, reinforcing the robustness of these findings.

We also disentangle competition’s mechanisms. The effect of hospital competition on non-technical healthcare quality is primarily driven by healthcare income, while the impact on technical healthcare quality is driven by healthcare demand and income. These mechanisms reveal critical insights into hospital behavior under market pressures. For technical quality, healthcare demand and income incentives suggest hospitals optimize resource allocation through cost-efficient service delivery while maintaining clinical standards to retain patients. In contrast, non-technical quality improvements stem solely from income-seeking behavior, as hospitals invest in communication strategies or environmental improvements to gain more income—a classic example of quality differentiation in competition models.

Our findings offer evidence regarding the impact of hospital competition on healthcare quality in China and inform evidence-based policies to optimize hospital competition, advancing progress toward SDG3’s targets for good health and well-being. Hospital competition can be an effective strategy in China’s New Healthcare Reform for improving healthcare quality and easing access to high-quality healthcare services. First, intensify hospital competition reforms by strengthening the foundational role of competition policies. It involves encouraging both private investment in hospitals and internal competition within public institutions, reducing direct resource allocation by the government, and leveraging hospital competition to safeguard healthcare quality. Second, account for the differential effects of competition across hospital tiers and service types. Promote hierarchical healthcare systems to optimize resource allocation and ensure rational patient distribution between primary and high-tier hospitals. Additionally, balance inpatient and outpatient service quality, capitalizing on the spillover effect of inpatient quality to enhance overall healthcare quality. Third, take a more active role of hospital competition in improving non-technical healthcare quality. Promote the efficient flow of healthcare resources to alleviate overcrowding in some public hospitals, enhancing patients’ satisfaction. Private hospitals should refine their strategies, focusing on improving distinctive and professional non-technical aspects to boost healthcare income.

## Data Availability

The original contributions presented in the study are included in the article/supplementary material, further inquiries can be directed to the corresponding author.

## References

[1] Gaynor M Propper C Seiler S. Free to choose? Reform, choice, and consideration sets in the English National Health Service. Am Econ Rev. (2016) 106:3521–57. doi: 10.1257/aer.20121532, PMID: 29553210

[2] Cooper Z Gibbons S Skellern M. Does competition from private surgical centres improve public hospitals’ performance? Evidence from the English National Health Service. J Public Econ. (2018) 166:63–80. doi: 10.1016/j.jpubeco.2018.08.002

[3] Mutter RL Wong HS Goldfarb MG. The effects of hospital competition on inpatient quality of care. Inquiry. (2008) 45:263–79. doi: 10.5034/inquiryjrnl_45.03.263, PMID: 19069009

[4] Timofeyev Y Goldenova V Mantaeva E Jakovljevic M. The Impact of Hospital Competition on the Quality of Care in Europe: A Systematic Review. Healthcare. (2024) 12:2218. doi: 10.3390/HEALTHCARE12222218, PMID: 39595417 PMC11593865

[5] Yip W Hsiao W. Harnessing the privatisation of China’s fragmented health-care delivery. Lancet. (2014) 384:805–18. doi: 10.1016/S0140-6736(14)61120-X, PMID: 25176551 PMC7159287

[6] Lu LY Lin XJ Pan J. Heterogeneous effects of hospital competition on inpatient expenses: An empirical analysis of diseases grouping basing on conditions’ complexity and urgency. BMC Health Serv Res. (2021) 21:1322. doi: 10.1186/S12913-021-07331-1, PMID: 34893077 PMC8662870

[7] Liu Y. Understanding patient choice of health care facilities in China. Rotterdam: Erasmus University Rotterdam (2021).

[8] Kessler DP McClellan MB. Is hospital competition socially wasteful? Q J Econ. (2000) 115:577–615. doi: 10.1162/003355300554863

[9] Brekke KR Siciliani L Straume OR. Competition and waiting times in hospital markets. J Public Econ. (2008) 92:1607–28. doi: 10.1016/j.jpubeco.2008.02.003

[10] Moscelli G Gravelle H Siciliani L. Hospital competition and quality for non-emergency patients in the English NHS. Rand J Econ. (2021) 52:382–414. doi: 10.1111/1756-2171.12373

[11] Young M. (2016). Private vs. Public Healthcare in South Africa. Honors Theses 2741. Available at: https://scholarworks.wmich.edu/honors_theses/2741 (Accessed May 1, 2025).

[12] Selvaraj S Karan AK Srivastava S Bhan N Mukhopadhyay I. India health system review. New Delhi: World Health Organization Regional Office for South-East Asia (2022).

[13] Santos FPD Merhy EE. Public regulation of the health care system in Brazil: a review. Interface. (2006) 10:25–41. doi: 10.1590/S1414-32832006000100003

[14] Pan J Qin XZ Li Q Messina JP Delamater PL. Does hospital competition improve health care delivery in China? China Econ Rev. (2015) 33:179–99. doi: 10.1016/j.chieco.2015.02.002

[15] Wang YX Castelli A Cao Q Liu D. Assessing the design of China’s complex health system - concerns on equity and efficiency. Health Policy Open. (2020) 1:100021. doi: 10.1016/j.hpopen.2020.100021, PMID: 37383318 PMC10297750

[16] Lin XJ Jian WY Yip W Pan J. Perceived competition and process of care in rural China. Risk Manag Healthc Policy. (2020) 13:1161–73. doi: 10.2147/RMHP.S258812, PMID: 32884377 PMC7439494

[17] Tsai TC Joynt KE Orav EJ Gawande AA Jha AK. Variation in surgical-readmission rates and quality of hospital care. N Engl J Med. (2013) 369:1134–42. doi: 10.1056/NEJMsa1303118, PMID: 24047062 PMC4107655

[18] Kruk ME Gage AD Joseph NT Danaei G García-Saisó S Salomon JA. Mortality due to low-quality health systems in the universal health coverage era: a systematic analysis of amenable deaths in 137 countries. Lancet. (2018) 392:2203–12. doi: 10.1016/S0140-6736(18)31668-4 30195398 PMC6238021

[19] Chen CC Cheng SH. Hospital competition and patient-perceived quality of care: evidence from a single-payer system in Taiwan. Health Policy. (2010) 98:65–73. doi: 10.1016/j.healthpol.2010.06.022, PMID: 20650538

[20] Beaulieu ND Dafny LS Landon BE Dalton JB Kuye I McWilliams JM. Changes in quality of care after hospital mergers and acquisitions. N Engl J Med. (2020) 382:51–9. doi: 10.1056/NEJMsa1901383, PMID: 31893515 PMC7080214

[21] Wang Y Chen H. The impact of the implementation of hierarchical medical system on population health: evidence from China. Front Public Health. (2024) 12:1402832. doi: 10.3389/fpubh.2024.1402832, PMID: 38846612 PMC11153785

[22] Zhang XX Zimmerman A Zhang YY Ogbuoji O Tang SL. Rapid growth of private hospitals in China: emerging challenges and opportunities to health sector management. Lancet Reg Health West Pac. (2024) 44:100991. doi: 10.1016/j.lanwpc.2023.100991 38156262 PMC10753080

[23] Guan X Qi L Liu L. Controversy in public hospital reforms in China. Lancet Glob Health. (2016) 4:e240–14. doi: 10.1016/S2214-109X(16)00041-3, PMID: 27013311

[24] Qiu L Yu Y Liu J Hu H Wang Y. For-profit medical institutions in China: current status analysis and comparison with non-profit ones. J Pharm Health Serv Res. (2014) 5:37–47. doi: 10.1111/jphs.12047

[25] Eggleston K Lu MS Li CD Wang J Yang Z. Comparing public and private hospitals in China: evidence from Guangdong. BMC Health Serv Res. (2010) 10:1–11. doi: 10.1186/1472-6963-10-76, PMID: 20331886 PMC2858143

[26] Gao X Ying J Zhang Y. Study on the Influence of Private Hospital Advertising on Consumers’ Attitude to Brand. In 4th International Conference on Culture, Education and Economic Development of Modern Society (ICCESE 2020). Dordrecht: Atlantis Press. (2020): 1598–1603.

[27] Tang CX Zhang YC Chen LP Lin YQ. The growth of private hospitals and their health workforce in China: a comparison with public hospitals. Health Policy Plan. (2014) 29:30–41. doi: 10.1093/heapol/czs130, PMID: 23335465

[28] Wang C Chen YH. Reimbursement and hospital competition in China. Econ Res Ekon Istraž. (2017) 30:1209–23. doi: 10.1080/1331677X.2017.1340177, PMID: 40360821

[29] Zhong H. Effect of patient reimbursement method on health-care utilization: evidence from China. Health Econ. (2011) 20:1312–29. doi: 10.1002/hec.1670 20882574

[30] Gile PP van de Klundert J Buljac-Samardzic M. Strategic human resource management and performance in public hospitals in Ethiopia. Front Public Health. (2022) 10:915317. doi: 10.3389/fpubh.2022.915317, PMID: 36339178 PMC9632433

[31] Gile PP van de Klundert J Buljac-Samardzic M. Human resource management in Ethiopian public hospitals. BMC Health Serv Res. (2022) 22:763. doi: 10.1186/s12913-022-08046-7, PMID: 35689209 PMC9188153

[32] Paauwe J Ferndale E. Strategy, HRM, and performance: a contextual approach. 2nd ed. Oxford: Oxford University Press (2017).

[33] Johnson P Szamosi LT. Human resource management: a critical approach. Second ed. Milton Park: Routledge (2018).

[34] Assefa T Mariam DH Mekonnen W Derbew M. Health system’s response for physician workforce shortages and the upcoming crisis in Ethiopia: a grounded theory research. Hum Resour Health. (2017) 15:86. doi: 10.1186/s12960-017-0257-5, PMID: 29282069 PMC5745790

[35] Prineas S Mosier K Mirko C Guicciardi S, Non-technical skills in healthcare. In Donaldson L Ricciardi W Sheridan S Tartaglia R, (eds) Textbook of patient safety and clinical risk management. Cham: Springer, (2021) 413–434. 36315753

[36] Alhassan RK Duku SO Janssens W Nketiah-Amponsah E Spieker N van Ostenberg P. Comparison of perceived and technical healthcare quality in primary health facilities: implications for a sustainable National Health Insurance Scheme in Ghana. PLoS One. (2015) 10:e0140109. doi: 10.1371/journal.pone.0140109, PMID: 26465935 PMC4605650

[37] Scott J Morales DR McRitchie A Riviello R Smink D Yule S. Non-technical skills and health care provision in low- and middle- income countries: a systematic review. Med Educ. (2016) 50:441–55. doi: 10.1111/medu.12939, PMID: 26995483

[38] Jamalabadi S Winter V Schreyögg J. A systematic review of the association between hospital cost/price and the quality of care. Appl Health Econ Health Policy. (2020) 18:625–39. doi: 10.1007/s40258-020-00577-6, PMID: 32291700 PMC7518980

[39] Stanton P. Managing the healthcare workforce: cost reduction or innovation. Aust Health Rev. (2002) 25:92–8. doi: 10.1071/ah020092, PMID: 12404971

[40] Zhao YH Strauss J Yang GH Giles J Hu PF Hu YS. China Health and Retirement Longitudinal Study: 2011–2012 National Baseline User’s Guide. Beijing: National School of Development, Peking University (2013).

[41] Zhao YH Hu YS Smith JP Strauss J Yang GH. Cohort profile: the China health and retirement longitudinal study (CHARLS). Int J Epidemiol. (2014) 43:61–8. doi: 10.1093/ije/dys203, PMID: 23243115 PMC3937970

[42] Shu JL Xie CS Gao L Wang ZZ Ren QF Sun JH. Association of depressive symptoms with non-fatal cardiovascular disease in middle-aged and elderly patients with hypertension: a cohort study from China. BMJ Open. (2025) 15:e087905. doi: 10.1136/bmjopen-2024-087905, PMID: 40180414 PMC11966951

[43] Chen J Lin ZC Li LA Li J Wang YY Pan Y. Ten years of China’s new healthcare reform: a longitudinal study on changes in health resources. BMC Public Health. (2021) 21:2272–2. doi: 10.1186/s12889-021-12248-9, PMID: 34903184 PMC8670033

[44] Asclepius. Annual Report on China’s Hospitals Competitiveness (2015). Beijing: Social Sciences Academic Press (2016).

[45] Asclepius. Annual Report on China’s Hospitals Competitiveness (2018–2019). Beijing: Social Sciences Academic Press (2019).

[46] Donabedian A. The Quality of Care. JAMA. (1988) 260:1743–8. doi: 10.1001/jama.1988.03410120089033 3045356

[47] Geldsetzer P Haakenstad A James EK Atun R. Non-technical health care quality and health system responsiveness in middle-income countries: a cross-sectional study in China, Ghana, India, Mexico, Russia, and South Africa. J Glob Health. (2018) 8:020417. doi: 10.7189/jogh.08.020417, PMID: 30356805 PMC6189548

[48] Jiang QL Tian F Liu ZM Pan J. Hospital competition and unplanned readmission: Evidence from a systematic review. Risk Manag Healthc Policy. (2021) 14:473–89. doi: 10.2147/RMHP.S290643, PMID: 33574721 PMC7873024

[49] Dimick JB Ghaferi AA. Hospital readmission as a quality measure in surgery. JAMA. (2015) 313:512–3. doi: 10.1001/jama.2014.14179, PMID: 25647207

[50] Reader T Flin R Lauche K Cuthbertson BH. Non-technical skills in the intensive care unit. Br J Anaesth. (2006) 96:551–9. doi: 10.1093/bja/ael067, PMID: 16567346

[51] Gavurova B Dvorsky J Popesko B. Patient satisfaction determinants of inpatient healthcare. Int J Environ Res Public Health. (2021) 18:11337. doi: 10.3390/IJERPH182111337, PMID: 34769856 PMC8582779

[52] Strumann C Geissler A Busse R Pross C. Can competition improve hospital quality of care? A difference-in-differences approach to evaluate the effect of increasing quality transparency on hospital quality. Eur J Health Econ. (2022) 23:1229–42. doi: 10.1007/S10198-021-01423-9, PMID: 34997865 PMC9395484

[53] Cookson R Dusheiko M Hardman G Martin S. Competition and Inequality: Evidence from the English National Health Service 1991-2001. J Publ Adm Res Theor. (2010) 20:i181–205. doi: 10.1093/jopart/muq021

[54] Yang XS Liu HL Liu RM. Market structure, medical arms race and health expense growth: An empirical analysis based on the cross-province data. Chin Health Econ. (2014) 33:40–2. doi: 10.7664/CHE20140712

[55] Meng QY. Competition in medical care: market structure, competitiveness and effects. Chin Health Econ. (2003) 22:30–3.

[56] Dranove D Lindrooth R White WD Zwanziger J. Is the impact of managed care on hospital prices decreasing? J Health Econ. (2008) 27:362–76. doi: 10.1016/j.jhealeco.2007.05.004, PMID: 18215433

[57] Gaynor M Moreno-Serra R Propper C. Death by market power: Reform, competition, and patient outcomes in the National Health Service. Am Econ J Econ Policy. (2013) 5:134–66. doi: 10.1257/pol.5.4.134

[58] Lin XJ Cai M Fu Q He K Jiang TY. Does hospital competition harm inpatient quality? Empirical evidence from Shanxi, China. Int J Environ Res Public Health. (2018) 15:2283. doi: 10.3390/ijerph15102283, PMID: 30336629 PMC6210984

[59] Lu LY Pan J. Does hospital competition lead to medical equipment expansion? Evidence on the medical arms race. Health Care Manag Sci. (2021) 24:582–96. doi: 10.1007/S10729-020-09529-X, PMID: 33411086

[60] Gravelle H Santos R Siciliani L. Does a hospital’s quality depend on the quality of other hospitals? A spatial econometrics approach. Reg Sci Urban Econ. (2014) 49:203–16. doi: 10.1016/j.regsciurbeco.2014.09.005, PMID: 25843994 PMC4375725

[61] Moscelli G Gravelle H Siciliani L. Market structure, patient choice and hospital quality for elective patients. York, UK: CHE Research Paper, Centre for Health Economics, University of York (2016). 139 p.

[62] Dzampe AK Takahashi S. Competition and quality of care under regulated fees: Evidence from Ghana. Health Econ Rev. (2022) 12:57. doi: 10.1186/S13561-022-00406-7, PMID: 36355234 PMC9647994

[63] Moscelli G Gravelle H Siciliani L Gutacker N. The effect of hospital ownership on quality of care: Evidence from England. J Econ Behav Organ. (2018) 153:322–44. doi: 10.1016/j.jebo.2018.05.009

[64] Liu X Su C Shao H. Intergenerational succession and excess control of family board seats. Econ Res J. (2021) 15:111–29.

[65] Rogowski J Jain AK Escarce JJ. Hospital competition, managed care, and mortality after hospitalization for medical conditions in California. Health Serv Res. (2007) 42:682–705. doi: 10.1111/j.1475-6773.2006.00631.x, PMID: 17362213 PMC1955358

[66] Pauly MV McGuire TG Barros PP. Handbook of health economics. Amsterdam: Elsevier (2012).

[67] Yu HY Wang PP Zheng H Luo JF Liu J. Impacts of congestion on healthcare outcomes: an empirical observation in China. J Manag Anal. (2020) 7:344–66. doi: 10.1080/23270012.2020.1731720, PMID: 40360821

[68] Li X Lu JP Hu S Cheng KK De Maeseneer J Meng QY. The primary health-care system in China. Lancet. (2017) 390:2584–94. doi: 10.1016/S0140-6736(17)33109-4, PMID: 29231837

